# Tentative Application of a Streamlined Protocol to Determine Organ-Specific Regulations of Deiodinase 1 and Dehalogenase Activities as Readouts of the Hypothalamus-Pituitary-Thyroid-Periphery-Axis

**DOI:** 10.3389/ftox.2022.822993

**Published:** 2022-03-21

**Authors:** Kostja Renko, Helena Kerp, Janina Pape, Eddy Rijntjes, Tanja Burgdorf, Dagmar Führer, Josef Köhrle

**Affiliations:** ^1^ German Federal Institute for Risk Assessment (BfR), German Centre for the Protection of Laboratory Animals (Bf3R), Berlin, Germany; ^2^ Charité, Universitätsmedizin Berlin, Corporate Member of Freie Universität Berlin and Humboldt-Universität zu Berlin, Institut für Experimentelle Endokrinologie, Berlin, Germany; ^3^ Department of Endocrinology, Diabetes and Metabolism, University Hospital Essen, University of Duisburg-Essen, Duisburg, Germany

**Keywords:** thyroid hormone system, deiodinase, dehalogenase, biomarker, activity assay, iodine status, regulatory testing, endocrine disruption

## Abstract

In animal studies, both in basic science and in toxicological assessment of potential endocrine disruptors, the state of the thyroid hormone (TH) axis is often described and defined exclusively by the concentrations of circulating THs and TSH. Although it is known that the local, organ-specific effects of THs are also substantially regulated by local mechanisms such as TH transmembrane transport and metabolism of TH by deiodinases, such endpoint parameters of the axis are rarely assessed in these experiments. Currently developed *in vitro* assays utilize the Sandell-Kolthoff reaction, a photometric method of iodide determination, to test the effect of chemicals on iodotyrosine and iodothyronine deiodinases. Furthermore, this technology offers the possibility to determine the iodine content of various sample types (e.g., urine, *ex vivo* tissue) in a simple way. Here, we measured deiodinase type 1 and iodotyrosine dehalogenase activity by means of the Sandell-Kolthoff reaction in *ex vivo* samples of hypo- and hyperthyroid mice of two age groups (young; 3 months and old; 20 months). In thyroid, liver and kidney, organ-specific regulation patterns emerged across both age groups, which, based on this pilot study, may serve as a starting point for a deeper characterization of the TH system in relevant studies in the future and support the development of Integrated Approach for Testing and Assessment (IATA).

## Introduction

Thyroid hormones (THs) exert multiple effects on the mammalian organism. Their tightly regulated biosynthesis and action are essential in the developing organism and disturbances can, for example, harm neurological development (Gilbert et al., 2020). However, THs also have multiple regulatory roles in the adult organism and affect differentiation, growth, metabolism, as well as behaviour.

Systemic TH concentrations, TSH as a sensitive indicator of the feedback loop, and thyroid histology are traditional *ex vivo* markers of chronic and acute events affecting the Hypothalamus-Pituitary-Thyroid-Periphery (HPTP)-axis. The current picture of TH-related mechanisms has expanded over the last decades. Local mechanisms of hormonal regulation were described, including TH trans-membrane or trans-barrier (e.g., blood-brain-barrier or brain-placenta-barrier) transport, local TH activation or inactivation by deiodination, elimination *via* sulfation or glucuronidation, and a complex interplay between different isoforms of TH-receptors (TR) (Murk et al., 2013) as well as canonical and non-canonical TR signalling. For most of these local mechanisms, a substantial impact on proper TH action was impressively shown by respective pathologies due to (in-)activating mutations in patients (Afink et al., 2008; Dumitrescu et al., 2010; van Geest et al., 2021) or genetically modified mice (Fujisawa et al., 2020; Salveridou et al., 2020; Morte et al., 2021).

Disruption of the TH system can result from disease, but essential components of the HPTP-axis have recently become the focus of studies evaluating the disruptive effects of environmental chemicals. Such compounds that directly interfere with the HPTP axis (apart from other targets in the endocrine system) and elicit adverse health effects are defined as endocrine disruptors (ED) and thus fall under regulatory restrictions. To define a chemical compound as ED, a causal link must be established between an endocrine molecular mechanism and an adverse outcome (Noyes et al., 2019). While several molecular mechanisms can now be well tested and identified with appropriate *in vitro* assays, establishing a cause-effect chain usually still requires *in vivo* data (Kortenkamp et al., 2020). In chemical testing such conclusive data on key events and adverse effects are often derived from defined standard animal tests, as described in globally accepted test guidelines (TG, e.g., OECD TG No. 408 or 414). However, the number of biomarkers and quantifiable key events included in defined animal protocols for testing ED effects on the HPTP axis is limited. An expansion of this spectrum of optional parameters, especially including read-outs that can be retrospectively measured from preserved tissue samples, would be useful and might contribute to the refinement and sustained analysis of *in vivo* TG experiments.

We recently published several *in vitro* assays to test potential EDs for their disruptive effects on processes affecting local TH availability, including protocols for all three iodothyronine deiodinase isoenzymes (DIO1-3) (Renko et al., 2012, 2015), the iodotyrosine dehalogenase (DEHAL1) (Renko et al., 2016) and the TH transmembrane transporter MCT8 (Jayarama-Naidu et al., 2015). By using the Sandell-Kolthoff-reaction (Sandell and Kolthoff, 1934) as a common analytical approach to quantify released or transported iodide, we configured a comprehensive and well-accepted technology for various *in vitro* assays related to the HPTP-axis. In fact, these assays are already being used internationally, and screenings for potential ED based on them have been successfully performed (Dong and Wade, 2017; Hornung et al., 2018; Olker et al., 2021). In principle, these assays are not only applicable to *in vitro* assays using recombinant enzymes or transgenic cell lines but also suitable for *ex vivo* material from animal experiments.

Utilizing a clearly defined rodent model for hypo- and hyperthyroidism, we aimed to evaluate the usefulness of Dio1 activity, Dehal1 activity, thyroid iodine content and urinary iodine concentration as meaningful *ex vivo* parameters, available through this platform technology to obtain additional local and organ-specific readouts of the TH status. Both enzymatic activities were shown to be regulated by TH serum levels and iodine status and represent crucial functions in TH metabolism, iodine recycling and retention. In this regard, established animal protocols of induction of hypo- and hyperthyroidism in rodents can serve as a starting point to characterize the consequences of disruption of the HPTP-axis by external factors, like xenobiotics and to identify new biomarkers/assessment parameters for animal experiments. A pilot setup for the analysis of various relevant enzymatic activities and parameters from thyroid, liver and kidney was developed to extend the number of accessible local parameters with modest and technically feasible effort, avoiding the use of radioactively labelled molecules and using a microtiter format to optimize speed and handling.

Disorders of the HPTP axis may lead to adverse effects mediated by alterations of the thyroid hormone system independent of structural or functional changes of the thyroid gland. Dietary restriction of specific trace elements (Iodine and Selenium) can lead to isolated effects on functions of the thyroid or target organs, as demonstrated in a rodent experiment (Lossow et al., 2021). While iodine deficiency resulted in an activated thyroid with increased activities of peroxidase and deiodinases and changes in circulating T4 levels without substantial effects in liver and kidney, Se deficiency exclusively negatively affected deiodinase activities in these target organs. In a recent publication (Kerp et al., 2019), chronic hypo- and hyperthyroidism in mice, induced by antithyroid agents (perchlorate and methimazole (MMI)) or T_4_ injection, respectively, was used to study age-dependence of TH-driven effects on brain function and behaviour. Noteworthy, this experimental setup establishes a link between several molecular initiating events (MIE; suppression of sodium-iodide-symporter (NIS) function, inhibition of thyroperoxidase activitiy (TPO)) to an adverse outcome, i.e. neurological phenotype such as declined motor activity and elevated anxiety levels in hypothyroid animals. To gain further insight here, samples from a comparable animal experiment were used, including hyperthyroid (HYPER) and hypothyroid (HYPO) animals of two different ages (young (3 months, Y) and aged (20 months, O), to explore potential TH status biomarkers for future use and to develop a strategy of measurement and evaluation.

## Materials and Methods

### Materials

All chemicals were of analytical grade and obtained from Merck or Sigma-Aldrich. Dibromotyrosine (DBT) and monoiodotyrosine (MIT) were from Tokyo Chemical Industry. NADPH tetrasodium salt was from Carl Roth GmbH. Dowex W50 X2 was acquired from Sigma-Aldrich and equilibrated in acetic acid (10%). The vacuum plate prep manifold was from SUPELCO, and 96-deep well plates as well as filter plates for Dowex-columns were taken from the Eppendorf Perfectprep Plasmid 96 Vac Direct Bind kit as described before (Renko et al., 2012).

### Mice

Male wildtype C57BL/6JRj mice (Janvier Labs, France) aged 3 months (young, Y) and 20 months (old, O) were used for experiments on thyroid dysfunction. All mice were single-housed in individually ventilated cages in temperature (23 ± 1°C) and light (inverse 12:12-h light–dark cycle)-controlled conditions. Chronic hyperthyroidism (HYPER) was induced by adding 1 μg/ml T_4_ to the drinking water [T_4_ was dissolved in 40 mM NaOH and 0.1% bovine serum albumin (BSA)]. For induction of chronic hypothyroidism (HYPO), animals were fed a low-iodine diet (MD.1571, Envigo, United States) and received drinking water supplemented with 0.02% methimazole, 0.5% perchlorate and 0.3% saccharine as sweetener (LoI/MMI/ClO_4_^−^) (Rakov et al., 2016). Control and hyperthyroid animals were fed a control diet (low iodine diet with added potassium iodide). After a treatment period of 3 weeks, mice were sacrificed and the urine, liver, kidney and thyroid gland collected. Animal handling was carried out by the same experimenter to minimize stress level of mice. All animal experiments were performed in accordance with the German regulations for Laboratory Animal Science (GVSOLAS) and the European Health Law of the Federation of Laboratory Animal Science Associations (FELASA). The protocols for animal studies were approved by the Landesamt für Natur, Umwelt und Verbraucherschutz Nordrhein-Westfalen, Germany.

### Sample Preparation

Dissected tissue was snap-frozen in liquid nitrogen and stored at −80°C until use. For all subsequent sample preparations, the liver and kidney tissue were powdered in the frozen state and the number of analyzed samples per group and tissue was chosen upon availability. Tissue powder was mixed with 500 µl homogenization buffer (250 mM D-Sucrose, 20 mM Hepes, 1 mM EDTA, pH 7.4) and disrupted by sonification (2*10 pulses, 0.6 s at 200 W; B. Braun Biotech). Protein concentration from resulting homogenates was determined by Bradford assay using IgG as standard (BioRad), and homogenates were adjusted to a defined concentration. In the case of frozen thyroid lobes, tissue was homogenized *via* micro pestle (BioRad) in Tris-HCl (10 mM, pH 7.0). Resulting homogenate was subdivided for enzyme determination and iodine measurement, as shown in Figure 3A.

### Deiodinase Assay

Deiodinase activity was measured *via* the Sandell-Kolthoff-based non-radioactive method, essentially as described before (Renko et al., 2012).

For each type of sample, a fixed amount of protein (thyroid 20 μg, liver or kidney 60 µg) was added to PCR tubes in a total volume of 50 µl in duplicates. Four samples per tissue were randomly chosen as background control by addition of propylthiouracil as a DIO1 inhibitor (1 mM, PTU) for later subtraction. The reaction was started by addition of 50 µl mastermix, resulting in desired final concentrations of phosphate buffer (100 mM, 1 mM EDTA, pH 6.8), DTT (40 mM) as cofactor and rT_3_ (10 µM) as substrate. After an incubation time of 2 h at 37°C under constant shaking, 75 µl of the reaction mixtures were applied to Dowex W50-X2–filled microtiter format column packages and released iodide eluted *via* vacuum after addition of 100 µl acetic acid (10%). Eluted iodide was measured in the Sandell-Kolthoff-reaction after further dilution. 50 µl of the respective dilution was placed in a microtiter plate (Techno Plastic Products AG) and reaction was started by adding cerium solution (25 mM (NH_4_)_4_Ce(SO_4_)_4_ and 0.5 M H_2_SO_4_) and arsenite solution (25 mM NaAsO_2_, 0.8 M NaCl, and 0.5 M H_2_SO_4_). Changes in absorption (OD at 416 nm) were determined at the reaction starting point and after 20 min and absolute activities were approximated by using a separately measured iodide standard curve for interpolation.

### Dehalogenase Assay

Dehalogenase/Dehal1 activity was measured *via* the Sandell-Kolthoff-based non-radioactive method, essentially as described before (Renko et al., 2016).

Using a fixed amount of protein (thyroid 20 μg, liver or kidney 140 µg), samples were applied to PCR tubes in a total volume of 50 µl in duplicates. Four additional samples per tissue were randomly chosen as background control by addition of DBT as specific inhibitor of Dehal1 activity for later subtraction. The reaction was started by addition of 50 µl mastermix to achieve the final assay conditions (100 mM KPO_4_ pH7, 200 mM KCl, 50 mM β-mercaptoethanol, 0.8 mM NADPH, 30 µM FAD, 10 µM MIT). After an incubation time of 4 h at 37°C under constant shaking, reaction mixtures were applied to Dowex—filled microtiter format column packages and released iodide eluted *via* vacuum after addition of 100 µl acetic acid (10%). Eluted iodide was measured in the Sandell-Kolthoff-reaction after further dilution (described above). Absolute activities were approximated by using a separately measured iodide standard curve for interpolation.

### Relative Iodine Determination

Thyroid tissue homogenate was adjusted to a protein concentration of 0.5 μg/μl. Subsequently, 25 µl of homogenate was mixed with 25 µl ammonium persulfate (APS, 1.3 M in ddH_2_O) and digested at 90°C for 1 h using a thermocycler.

Resulting homogenates were further diluted (5-fold and 20-fold for young and old animals respectively) and measured in the Sandell-Kolthoff-reaction (described above). To approximate the relative iodine content, a separately measured iodide standard curve for interpolation was used.

5 µl urine was digested with APS (f.c. 0.65 M) in a total volume of 50 μl at 90°C for 1 h. Resulting lysate was diluted (25 µl + 25 µl ddH_2_O) and measured in the Sandell-Kolthoff-reaction (described above).

### TH Measurements

Free thyroxine (FT4), and free thyronine (FT3) in serum were measured using commercial ELISA kits applied according to the manufacturer’s instructions (DRG Instruments GmbH, Germany; minimum detectable TH concentrations: 0.05 ng/dl for fT_4_, and 0.05 pg/ml for fT_3_). Serum samples with known TH concentrations were used as controls.

### Statistics

Data are presented as mean plus/minus SD and individual values as data points in the bar graphs. Statistical analyses were performed using the IBM SPSS statistics package (version 25). *p*-values <0.05 were considered significant, unless stated otherwise. Differences between the euthyroid control, HYPO- and HYPER-group were analysed using an ANOVA and a Bonferonni post-hoc test.

## Results

### Urinary Iodine

**FIGURE 1:**
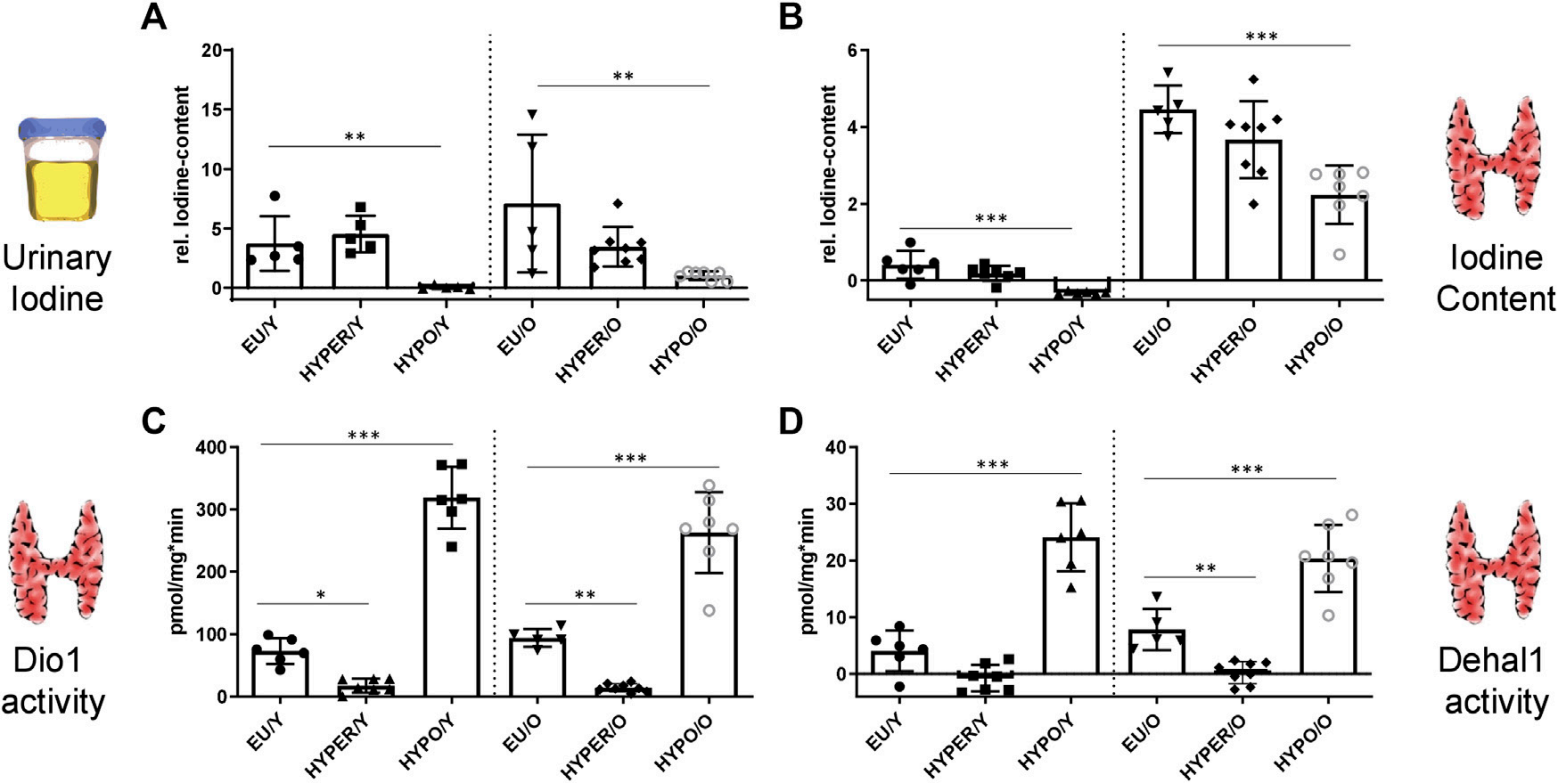
Urine iodine and thyroidal Dio1, Dehal1 and I-content is affected by HYPER and HYPO treatment; **(A)** As a marker of iodine intake and retention, spontaneous urine from mice is digested and measured for relative iodine content, showing reduced levels in the HYPO-group fed with iodine-deficient diet. **(B)** Iodine content of thyroids is strongly elevated in aged mice compared to young ones and reduced in the HYPO-groups, independent of age. **(C,D)** Thyroidal Dio1 and Dehal1 activities are decreased (HYPER) and increased (HYPO) compared to the control groups (EU), independent of age. [young groups (Y) on the left, old groups on the right (O)] All measurements used the Sandell-Kolthoff-reaction as readout. Significance is tested by ANOVA test followed by Bonferroni’s test. (*n* = 5–8/group); *p ≤ 0.05, **p ≤ 0.01, ***p ≤ 0.001.

Relative iodine content of spontaneous urine from mice was measured. While there was no difference between euthyroid and HYPER-animals, there was significantly reduced urinary iodine secretion in HYPO animals (Figure 1A) compared to the controls.

### Readouts in the Thyroid

The relative thyroid iodine content (Figure 1B) was reduced in the HYPO mice of either age. However, since older animals had already accumulated higher amounts of iodine in the thyroid gland complete depletion was not achieved even in the aged HYPO group. There was also a trend (not significant) for iodine content to be reduced in the HYPER animals.

Dio1 activity was shown to be a markedly regulated parameter in all treatment groups (Figure 1C). Compared with the euthyroid animals, activity was strongly suppressed in the HYPER groups and induced in the HYPO mice. These regulatory patterns were found in both age groups. Dehal1 shows an analogous regulation (Figure 1D).

### Enzyme Activities in Liver and Kidney

**FIGURE 2:**
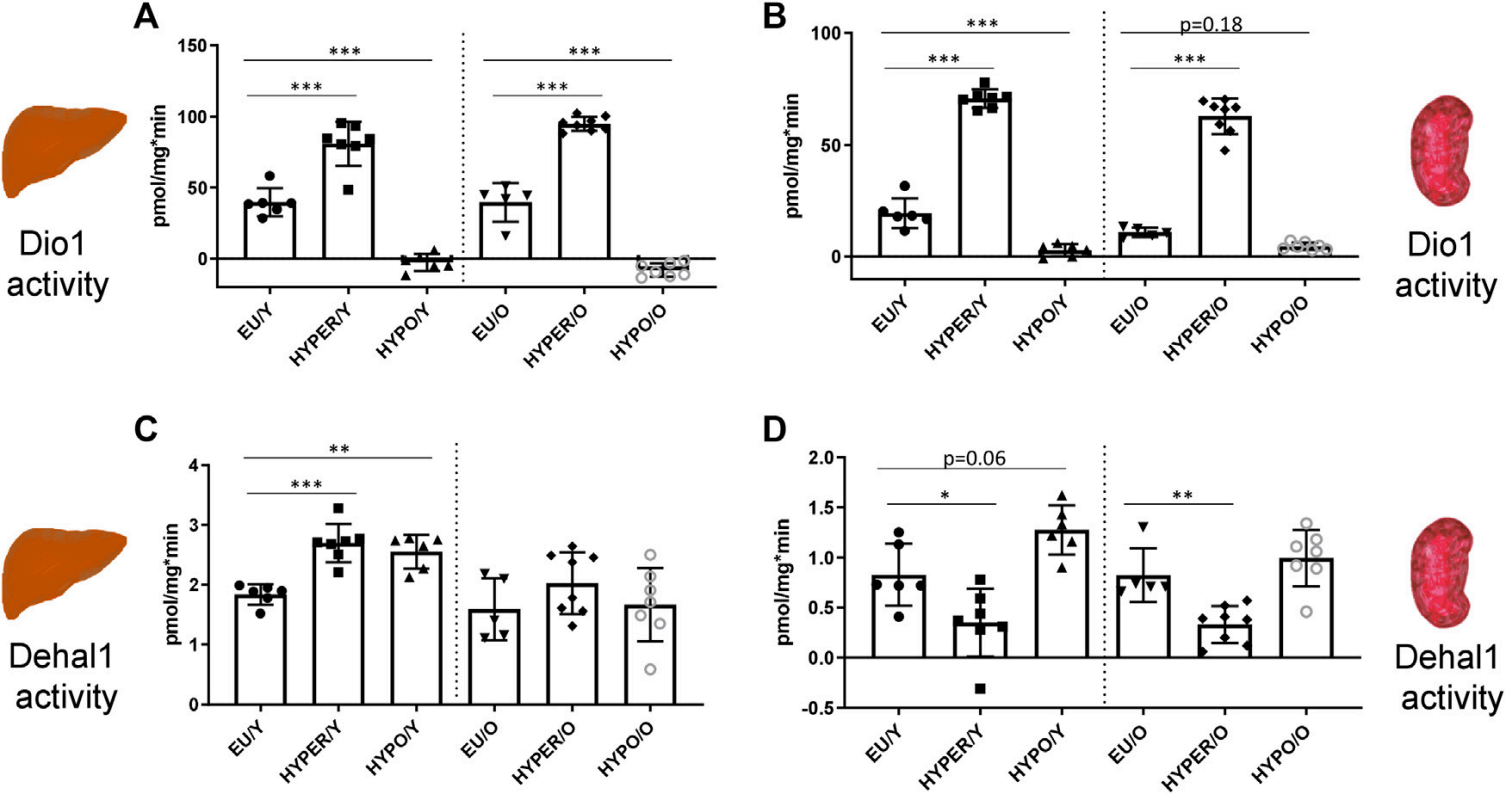
Deiodinating enzyme activities in liver and kidney; Dio1 activity is regulated in parallel in liver **(A)** and kidney **(B)** by HYPO and HYPER treatment. In contrast to thyroidal activity, in both organs Dio1 activity is strongly stimulated in HYPER animals and reduced in HYPO mice, independent of age. Dehal1 activity is just slightly changed in these organs, showing a slight but still significant induction in HYPER and HYPO groups in the liver **(C)**, exclusively in young animals. In the kidney **(D)**, HYPO treatment leads to reduced activity levels in both, young and aged mice [young groups (Y) on the left, old groups on the right (O)]. All measurements used the Sandell-Kolthoff-reaction as readout. Significance is tested by ANOVA test followed by Bonferroni’s test. (*n* = 5–8/group); *p ≤ 0.05, **p ≤ 0.01, ***p ≤ 0.001.

Dio1 and Dehal1 activities were also measured in liver and kidney, representing TH target organs and sites of local TH action and iodine metabolism. In contrast to the thyroid, Dio1 activities in liver and kidney (Figures 2A,B) were induced in HYPER-animals and strongly reduced in HYPO-groups. This pattern was persistent in young and old mice. Dehal1 was found to be just slightly regulated in the liver (Figure 2C), showing a minimal induction in HYPO- and HYPER-mice, being significant only in young animals. Dehal1 was reduced in kidneys of HYPER-mice, independent of age (Figure 2D). Furthermore, there was a tendency towards elevated Dehal1 activity in kidneys of the young HYPO-group.

### Serum TH Concentrations

Free T3 and free T4 from serum samples were measured by applying commercial ELISAs, resulting in concentration ranges in accordance with published data (Rakov et al., 2016). While HYPER/Y and HYPER/O groups clearly showed a significant increase in both parameters compared to the respective control group, HYPO-groups were not significantly changed. (Supplementary Figure S1).

## Discussion

Over the last decades there is increasing concern, that environmental compounds might affect hormonal systems, thereby leading to disbalance in the human organism, potentially affecting fertility, developmental processes or even the health status of following generations. The HPTP-axis is of high interest here, first of all because the iodine-containing THs are mimicked by organic halogenated chemicals due to structural similarities. Furthermore, TH are essential for fundamental and partially irreversible processes of intrauterine development as well as various homeostatic processes in the adult organism. Chemicals affecting this fine-tuned system might interfere with brain development, processes of cell differentiation in general or energy metabolism. Chemical testing in the past, but also nowadays, still depends on experimental protocols using rodent models to define and assess the hazard potential of respective chemicals. Future concepts or testing strategies to complement and even replace current *in vivo* TGs by *in vitro* testing still require robust data from the *in vivo* situation to qualify such *in vitro* assays as reliable and relevant. So far, no *in vitro* thyroid assay has been formally validated even though a number of assays has already been described and assessed in an OECD scoping document in 2014 (OECD, 2014). Based on recombinant enzymes, the activity of Dio1 and Dehal1 has been used as an important endpoint in the context of screening chemicals for TH system disruption (Hornung et al., 2018; Olker et al., 2021) but local effects *in vivo* have not been systematically addressed. With focus on the HPTP-axis, neglecting the local processes in target tissues that eventually define the health harm, such TG animal experiments in rodent models are quite limited in information regarding the number and complexity of parameters measured. Such an analysis usually does not exceed determination of circulating hormones and phenotypic evaluation of the thyroid gland itself (weight, histopathology). The OECD “Conceptual Framework for Testing and Assessment of Endocrine Disrupters” (OCDE, 2018) includes in levels 3–5 several *in vivo* study TGs dedicated to assess mammalian toxicity. Level 3 comprises *in vivo* assays that generate data on the affected mechanisms, whereas level 4 assays allow statements about adverse effects based on relevant endpoints. Level 5 comprises *in vivo* models that allow comprehensive statements regarding adverse effects and relevant endpoints over a longer period of time and thus different developmental stages of the organ. For example, a repeated dose 28-days study (OECD TG No. 407) and repeated dose 90-days study (OECD TG No. 408) are included in level 4. In their current versions both guidelines include thyroid histopathology in their list of required measurements. The updated TG No. 408 from 2018 also requires evaluation of thyroid weight, T_4_, T_3_ and TSH measurements, which were only optional in the TG No. 408 (and still are in TG 407), published in 2008.

A very recent study clearly demonstrates the diversity of phenotypic adversities, e.g., on brain development, even in situations of almost similar serum parameters (Gilbert et al., 2021). Here, obviously a more “local” perspective of hormonal regulation in different organs is needed, as recently demonstrated (Lossow et al., 2021). The presented “pilot” protocol opens the perspective to increase data output in given animal experimental protocols. The use of samples from various hierarchical levels of the HPTP axis (thyroid, liver, kidney) in combination with systemic parameters such as TH serum concentrations and inclusion of the underlying iodine status allows a more comprehensive picture of changes within the TH system, e.g., in pathology modelling or as a consequence of genetic modification or chemical exposure affecting specific or multiple key functions of the axis.

The protocol relies on the Sandell-Kolthoff reaction, applied to determine iodide-releasing enzymatic activities by non-radioactive iodide determination and iodine content of samples. Based on this simple colorimetric readout, Dio1 activity, Dehal1 activity and other accessible parameters were measured in samples from hypo- and hyperthyroid mice to evaluate a selection of biomarkers partially reflecting local TH-related functions for future animal studies. Noteworthy, Dio1 and Dehal1 activity have already been characterized as indicators of tissue-specific TH action and/or iodine status (Zavacki et al., 2005; Sun et al., 2015).

Because of its high Km-value in the µM-range, Dio1 is the only deiodinase isozyme accessible to the SK-reaction based non-radioactive iodine release assay method from tissue material. It contributes, together with Dio2, to the circulating levels of T_3_ by peripheral deiodination of thyroxine (Luongo et al., 2019). Furthermore, it exerts an important role in iodide recycling (Schneider et al., 2006). Due to its high affinity to TH-sulphates it has an major function in deiodinating these conjugates to prevent biliary secretion and loss of iodine (DiStefano et al., 1993). Being a selenoenzyme, its expression is depending on the availability of selenium as an essential, nutritional factor (Köhrle, 2000). Dio1 is positively regulated by T_3_ and was already suggested as sensitive marker of peripheral thyroid status in mice (Zavacki et al., 2005), being even superior over other T_3_-regulated transcripts in the liver. Moreover, Dio1 is strongly conserved across species (Orozco et al., 2012), thereby increasing its predictive value to extrapolate rodent studies towards the human situation.

Dehal1 activity found in thyroid, liver and kidney (and to a lesser extent in other organs) is essential to retain iodide in the organism. By releasing iodide from MIT and diiodotyrosine (DIT), by-products of TH biosynthesis, it prevents urinary loss by excretion of intact, iodinated amino acids, as it is seen in patients with respective mutations in the DEHAL1-gene (Moreno et al., 2008). Dehal1 is up-regulated by TSH-stimulation in the thyroid and decreases in response to chronic and acute iodide load (Solis-S et al., 2011). Furthermore, its enzymatic activity is decreased in the kidney of hyperthyroid mice (Renko et al., 2016) but not affected by iodine status here, in contrast to the thyroid (Sun et al., 2015).

The presented platform was applied using thyroid lobes from mice that usually do not exceed a total mass in the low mg-range. However, we could show that relative iodine content, Dio1 activity and Dehal1 activity can be assessed from a minimal amount of tissue, even limited to one lobe of a rodent model as mouse or rat, leaving the second lobe for histology.

For the thyroid, relative changes in total iodine content (Figure 1B) can be connected to the iodine uptake into the organism or, in particular, the gland itself. Together with the iodine measurements from spontaneous urine (Figure 1A), these parameters provide insight into the acute and chronic consequences of reduced iodide uptake into the organ, both by dietary limitation and by blocking the appropriate molecular mechanisms (in this case of NIS by NaClO_4_). The importance of thyroidal iodine content as a long-term, integral readout of iodine supply becomes apparent when comparing the different age groups. Albeit hypothyroidism is reflected (acutely) by altered tissue enzyme activities of Dio1 and Dehal1 in both age groups, iodine depletion in the thyroid tissue was less severe in elderly mice. Noteworthy, also the circulating TH concentrations in the aged HYPO mice were less affected.

In our study, the applied model of hypothyroidism, depleted iodine stocks lead to increased stimulation of the thyroid gland, probably through TSH increase and rise of thyroidal sensitivity towards TSH stimulation (Brabant et al., 1992). The lower iodine content in the thyroids of young animals of all groups probably results from the shorter lifespan before induction of hypothyroidism. Furthermore, reports on thyroid structure in aged rodents have described increasing numbers of inactive, “cold” follicles (Tamura and Fujita, 1981), probably unable to mobilize stored iodine/TH and potentially leading to a higher iodine content here. Thyroidal Dio1 and Dehal1 are stimulated in their activity in this situation in both age groups. Increased Dehal1 activity indicates activation of iodine recycling in the thyroid, especially in a situation of severe iodine deficiency (Solis-S et al., 2011; Lossow et al., 2021). Both activities appear to be clearly regulated by the activation state of the thyroid gland and partially opposite to their regulation in peripheral organs, where, at least for the Dio1, a parallel regulation to TH levels is evident.

Renal and hepatic Dio1 activity turns out to be a strongly regulated readout in hypo- and hyperthyroid animals as well. In HYPO animals, Dio1 activity was decreased in liver and kidney and increased in the HYPER groups, compared to the control animals respectively. Dio1 is a direct TH-target gene with a classical TH-responsive element in its promoter; its activity reflects the whole cascade of systemic and local mechanisms upstream of nuclear T_3_ receptor transactivation, suggesting its use as good indicator and biomarker for local TH action. Being accessible in liver and kidney samples, it can be measured from two organs that differ in their “position” within the TH system. Here the liver serves as a central organ not only affected by systemic TH, but also contributing to systemic TH circulation both by its high local deiodinase 1 activity and as major site of termination of TH action by conjugating phase II enzymes (van der Spek et al., 2017). Both organs are of comparatively large size, therefore enough sample material is usually available from animal experiments for analysis of numerous parameters. Because liver and kidney are often preserved as frozen specimen in all kinds of toxicological studies or basic science experiments, these enzymatic endpoint parameters open the perspective to expand the evaluation towards the HPTP axis even retrospectively.

**FIGURE 3:**
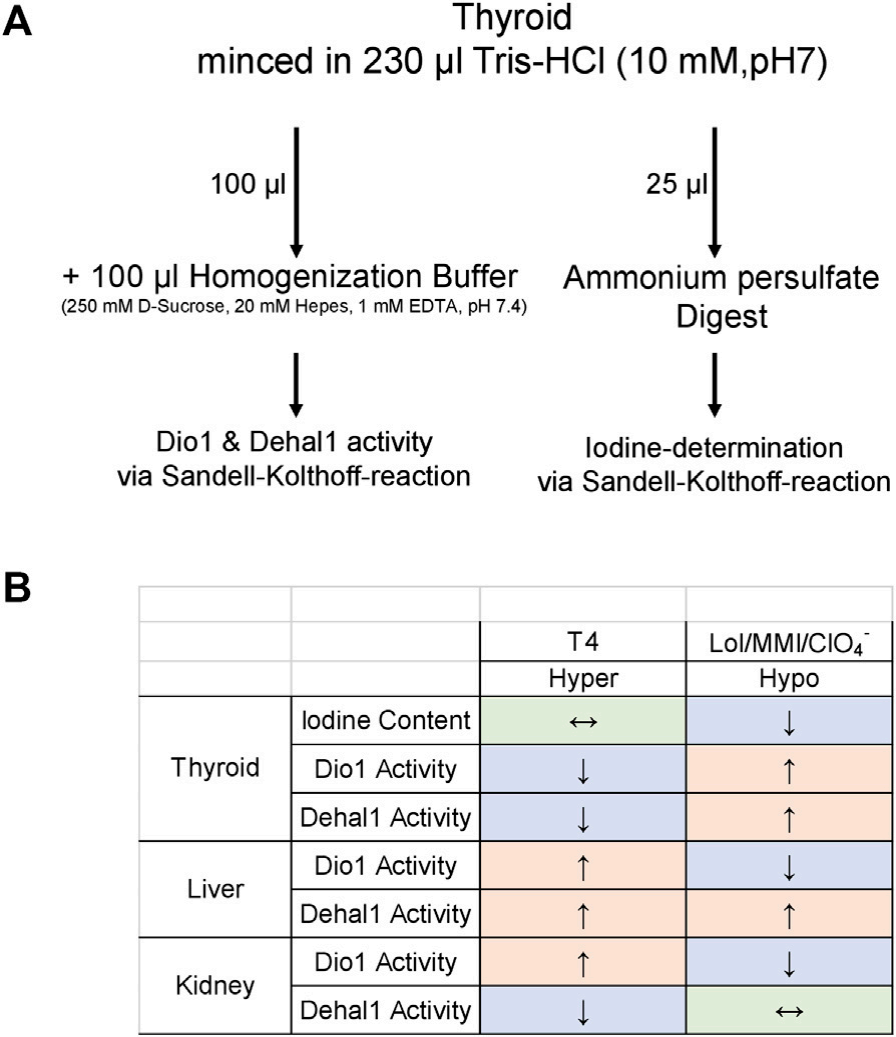
Organ-specific patterning of biomarkers; By utilizing a respective pilot protocol for sample preparation **(A)** and measurement, a patterning of readouts **(B)** including thyroidal iodine content, Dio1 and Dehal1 from thyroid, liver and kidney was generated. The suggested patterning of these readouts appears to be suitable to define situations of hypo- and hyperthyroidism. Its application and verification in future studies would be helpful to determine local TH status and to observe disruption of the TH system in toxicological studies. All readouts are accessible *via* Sandell-Kolthoff-based assays as unified readout platform technology.

In our study, most determined parameters in all investigated organs were changed in a characteristic way, generating a draft pattern of measurable potential biomarkers of hypo- and hyperthyroidism in rodent models for future verification (Figure 3B). A low basal level of absolute Dehal1 activity was detected compared to Dio1. To enhance sensitivity of this parameter, the assay setup may undergo optimization for future applications to gain higher signals for an improved detection. This can generally be achieved for both enzymatic assays by increasing the amount of protein per reaction, which will also help to omit calculated “negative activity values”, most likely resulting from incomplete inhibition of the background control samples.

Overall, while the suggested protocols are convenient in handling and avoid expensive equipment, they have some disadvantages that should be kept in mind. Compared to the radioactive version or protocols that directly detect educt and product, e.g., *via* LC-MS, the calculated total activities are extrapolated and do provide relative estimates rather than absolute values. Here, a higher degree of standardisation would be needed to allow transferability and reproducibility. Furthermore, determination of iodine content from urine or tissue is linked with an oxidation step with APS. The degree of iodine-release from different iodine-containing organic or inorganic forms is undetermined. Nevertheless, relative alterations within one experiment can obviously follow a meaningful pattern, as seen in this study. However, this versatile protocol doesn’t compete with labour- and resource-intensive classical, high-quality methods of elemental analysis and quantification.

Both, Dio1 and Dehal1, are coupled to changes in the hormonal axis, but in an individual manner, so that the parallel determination of these parameters offers an informative benefit. These activities are reflecting two separate local key functions in TH and iodine homeostasis that can be affected in a specific way by hormonal or nutritive changes, but also by environmental chemicals. Furthermore Dio1 and Dehal1 activity in peripheral tissues provide information on local concentrations of potential inhibitors thereby addressing additional key biological mechanisms and aspects of toxicokinetics. As recently demonstrated by a rodent model of dietary intervention, iodine-deficiency primary affects thyroid functions and activities, while other factors (here selenium-deficiency) lead to isolated Dio1 and Dehal1 changes in target organs (Lossow et al., 2021). This clearly demonstrates the added value of HPTP-axis dependent readouts separately addressing thyroidal and target organ status.

A recently suggested AOP-network (Noyes et al., 2019), connects chemical interactions with TH system molecular targets to downstream events and adverse outcomes. Inhibition of DIOs and DEHAL1 are discussed as important Molecular Initiating Events (MIE). In total, more than 25 MIEs are listed, that might upon chemical interference, lead or contribute to respective adverse changes of systemic or local TH concentrations and action, thereby potentially affecting e.g., fetal brain development and adult brain function among others (Gilbert et al., 2020; Affortit et al., 2021).

The AOP network for TH system disruption includes key functions of TH biosynthesis, but also local mechanisms to regulate TH action on the cellular level including cellular uptake, activation, inactivation and excretion. Interferences with local functions are not necessarily reflected by changes of systemic TH or TSH concentrations. Such a complex, endocrine network, spanning various cell types and organ systems with respective interfaces cannot be addressed by single *in vitro* assays but requires the development of IATAs or Defined Approaches in order to replace or reduce animal testing. *In chemico* or *in vitro* assays on single MIE or KE within the HPTP-system can only be one integral part of an IATA.

Maximising the output and adding novel endpoints to traditional toxicological *in vivo* studies will help to further elucidate the underlying AOP for TH system disruption and can inform an IATA in a weight-of-evidence approach. Furthermore, this data may help to address sex-specific aspects of the HPTP-axis, which are usually excluded *in vitro* but may be crucial in risk assessment strategies to define vulnerable groups. Our suggested protocol is a first attempt to expand the portfolio of biomarkers to local activities with organ-distinct regulation patterns (Figure 3B) that might help to interpret deflections of the HPTP-axis in more detail. Dio1 activity could be proposed as a hub within the AOP network, being a MIE by itself but also affected by hormonal action (TSH or TH) within the target tissue. Dehal1 activity as additional MIE in different organs, together with iodine content and urinary iodine excretion offer an interesting perspective on iodine status, retention and recycling (where Dio1 is also involved). As the iodine status of an organism is most likely a major determinant of vulnerability towards HPTP disruption this joint information might add another, highly relevant layer of information to respective studies and could help to strengthen the linkages between molecular targets and apical affects. Furthermore, comprehensive *in vivo* data including data on enzyme activity in different target organs from *in vivo* studies can be used to evaluate and improve predictivity of *in vitro* assays, especially more complex systems such as organ-on-a-chip, which are urgently needed to set up reference compound libraries and to improve the QSAR models for *in silico* predictions. The latter form an additional source of information that can be incorporated into integrated testing strategies.

An extensive analysis of *in vivo* samples, already available or generated in upcoming animal studies, is an important option to support and refine ongoing research on TH system-related pathologies and toxicological studies in the future. Further application of the suggested methods under conditions of toxicological studies will be needed to confirm their added value in situations of minor dysregulations of the thyroid hormone system.

## Data Availability

The raw data supporting the conclusions of this article will be made available by the authors, without undue reservation.
