# Supplementary material for: Tentative Application of a Streamlined Protocol to Determine Organ-Specific Regulations of Deiodinase 1 and Dehalogenase Activities as Readouts of the Hypothalamus-Pituitary-Thyroid-Periphery-Axis
Source: Front Toxicol. 2022 Mar 21;4:822993. doi: 10.3389/ftox.2022.822993 (PMC8978789; doi:10.3389/ftox.2022.822993)
Supplement: Supplementary file 1 [file DataSheet1.PDF]

Supplementary Figure 1

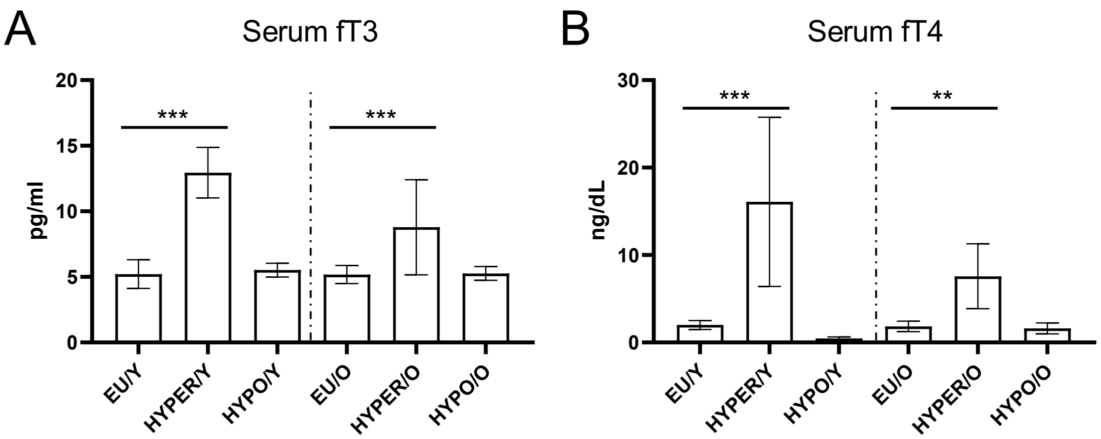

Suppl. Fig.1: Serum concentrations of fT3 and fT4 in EU-, HYPER- and HYPO-groups; Circulating concentrations of fT3 (A) and fT4 (B) were determined by commercial ELISA. While treatment of the HYPER-groups lead to a significant increase of both parameters, no significant changes were found in the HYPO-groups, tested by ANOVA test followed by Bonferroni's test. (n=6-11/group); $^{*}P \leq 0.05$, $^{**}P \leq 0.01$, $^{***}P \leq 0.001$.
